# Decoding of Pain Perception using EEG Signals for a Real-Time Reflex System in Prostheses: A Case Study

**DOI:** 10.1038/s41598-020-62525-7

**Published:** 2020-03-27

**Authors:** Zied Tayeb, Rohit Bose, Andrei Dragomir, Luke E. Osborn, Nitish V. Thakor, Gordon Cheng

**Affiliations:** 1 Institute for Cognitive Systems, Technical University of Munich, Arcisstraße 21, 80333 München, Germany; 2 N.1 Institute for Health, National University of Singapore, 28 Medical Dr. 05-COR, Singapore, 117456 Singapore; 3 Department of Bioengineering, University of Pittsburgh, 3700 O’Hara Street, Pittsburgh, PA 15261 USA; 4 Department of Biomedical Engineering, University of Houston, 3517 Cullen Blvd, Houston, TX 77204 USA; 5 Department of Biomedical Engineering, Johns Hopkins School of Medicine, 720 Rutland Ave, Baltimore, MD 21205 USA; 6 Research Exploratory Development, Johns Hopkins University Applied Physics Laboratory, 11100 Johns Hopkins Rd, Laurel, MD 20723 USA; 7 Department of Biomedical Engineering, National University of Singapore, Engineering Drive 3, 04-08, Singapore, 117583 Singapore

**Keywords:** Biomedical engineering, Electrical and electronic engineering

## Abstract

In recent times, we have witnessed a push towards restoring sensory perception to upper-limb amputees, which includes the whole spectrum from gentle touch to noxious stimuli. These are essential components for body protection as well as for restoring the sense of embodiment. Notwithstanding the considerable advances that have been made in designing suitable sensors and restoring tactile perceptions, pain perception dynamics and its decoding using effective bio-markers, are still not fully understood. Here, using electroencephalography (EEG) recordings, we identified and validated a spatio-temporal signature of brain activity during innocuous, moderately more intense, and noxious stimulation of an amputee’s phantom limb using transcutaneous nerve stimulation (TENS). Based on the spatio-temporal EEG features, we developed a system for detecting pain perception and reaction in the brain, which successfully classified three different stimulation conditions with a test accuracy of 94.66%, and we investigated the cortical activity in response to sensory stimuli in these conditions. Our findings suggest that the noxious stimulation activates the pre-motor cortex with the highest activation shown in the central cortex (Cz electrode) between 450 ms and 750 ms post-stimulation, whereas the highest activation for the moderately intense stimulation was found in the parietal lobe (P2, P4, and P6 electrodes). Further, we localized the cortical sources and observed early strong activation of the anterior cingulate cortex (ACC) corresponding to the noxious stimulus condition. Moreover, activation of the posterior cingulate cortex (PCC) was observed during the noxious sensation. Overall, although this is a single case study, this work presents a novel approach and a first attempt to analyze and classify neural activity when restoring sensory perception to amputees, which could chart a route ahead for designing a real-time pain reaction system in upper-limb prostheses.

## Introduction

**Figure 1:**
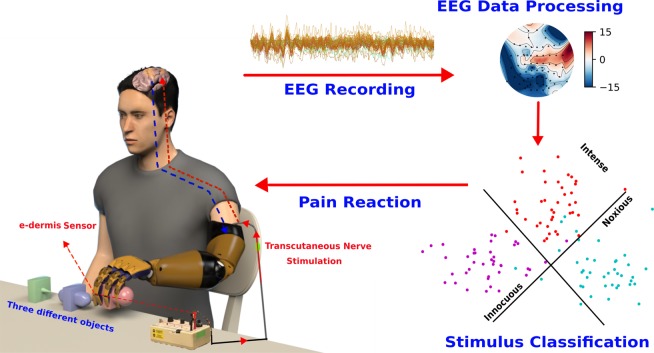
System implementation overview of a prosthetic arm that can restore the sense of touch and pain. An upper-limb amputee wears a prosthesis equipped with e-dermis sensors capable of measuring pressure and object curvature. Tactile sensations from innocuous to noxious, based on detected pressures at the fingertips, are conveyed to the user’s phantom hand through TENS on the residual limb. Brain responses are analyzed and decoded to understand the tactile sensory perception, including pain, and identify activated brain regions. Neural activity can be used to design a prosthesis that mimics natural pain withdrawal behavior in humans.

Nociception is commonly known as the sense of pain^1^. Specialized receptors called nociceptors that cover the skin and organs react to harmful chemical, mechanical and thermal stimuli^2^. Some of these microscopic pain receptors react to all kinds of noxious stimuli while others only react to specific pain like burning or pricking your finger on something sharp. Jolts of sudden pain activate the A-type fibers to send an electrical signal up to the spinal cord^3^. Pain signals then activate the thalamus, which relays the signal to the different brain regions^4^. Subsequently, the signal activates the somatosensory cortex which is responsible for physical sensations; the signals are then relayed to the frontal cortex where higher-order cognitive processing occurs, and finally to the limbic system, which is linked to emotions^5^. This pain processing network, along with pain reflex pathways in the spinal cord^6^, are considered of the utmost importance for body protection from damaging stimuli^7^. These insights into brain networks have therefore spurred research on unraveling the processes within the body that lead to the unpleasant sensation of pain^8^ and on understanding the pain perception mechanism in the brain^9^. Authors in^10^ investigated perceptual, motor, and autonomic responses to short noxious heat stimuli using electroencephalography (EEG) and confirmed that pain perception is subserved by a distinct pattern of EEG responses in healthy subjects. Functional magnetic resonance imaging (fMRI) was used in^11^ to demonstrate pain-related activation of the anterior cingulate cortex (ACC) and the posterior cingulate cortex (PCC) during transcutaneous electrical nerve stimulation (TENS) in healthy participants. A template of nociceptive brain activity that is sensitive to analgesic administration and suitable for clinical trials and research investigations was identified and validated in^12^. Furthermore, different somatosensory evoked potential (SEP) components and latency differences after stimulation of proximal and distal sites of the median nerves were studied and identified in eight healthy right-handed males^13^. Similarly, other previous studies showed that primary and secondary somatosensory cortices, insular cortex, anterior cingulate cortex (ACC), prefrontal cortex (PFC), and thalamus are activated during experimental pain stimuli^14–16^. Authors in^17^ showed the important role of the parietal lobe in pain perception and understanding. Notwithstanding the enormous number of studies on pain perception and brain responses to different painful stimuli using EEG and fMRI, most of these studies focused on studying brain responses in healthy subjects and have not investigated brain responses when perceiving the sense of pain in amputees nor in human-robot interaction settings^7^. It has, therefore, become imperative to study amputees’ brain activity when integrating the sense of touch and pain in their arm prostheses^7^. The core novelty and the main contribution of this paper reside in the use of non-invasive EEG activity to analyze somatosensory evoked responses recorded when receiving three different types of stimulations. These stimulations were chosen to convey different sensation profiles, ranging from pleasant to uncomfortable sensation. For that, we identified a brain activity template during innocuous (INNO), moderately intense (MOD) and noxious (NOX) stimulation of an amputee’s phantom hand delivered through a transcutaneous nerve stimulation system (TENS)^18^. Based on the identified spatio-temporal brain activity patterns, we developed an offline system for detecting pain reaction in the brain which can recognize the three stimulation conditions from recorded EEG responses by using effective spatio-temporal bio-markers to identify the different brain regions involved in noxious stimuli processing as well as latency responses for each stimulation condition. The overall goal of this study was to extend upon the work performed by Osborn *et al*.^7^, where the reflex system was implemented in the arm prosthesis and the amputee was not involved in the withdrawing reaction. This is thought to be of the utmost importance when designing a better bidirectional-control system between the human and the prosthesis, and hence increase the amputee’s sense of embodiment and the sense of ownership^19,20^. Additionally, detecting this perceived pain sensation and reaction would have an important role in protecting the prosthesis from being damaged by external stimuli^21^. To the best of our knowledge, even though the presented results are from a case study, this work is among the very few to investigate brain responses to different types of NOX and INNO stimuli and the first study to investigate and characterize spatio-temporal brain activities in amputees during a range of noxious and innocuous sensory feedback to the phantom hand, en route to designing a real-time withdrawal system in upper-limb prostheses. Extending upon the findings of the aforementioned studies, we also investigate attention and perceptual brain circuitry involved in the withdrawal reaction. An overview of the real-time withdrawal system in upper-limb prostheses is shown in Fig. 1.

## Results

### All stimulation conditions activate the parietal lobe and noxious stimulation activates the central motor cortex

**Figure 2:**
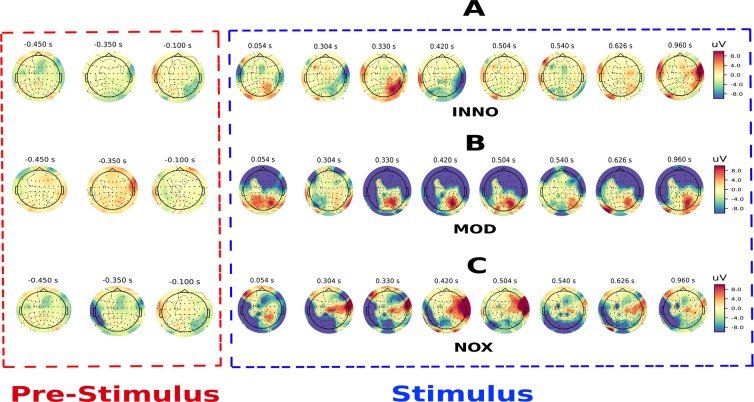
EEG topographic maps for NOX, MOD and the INNO stimulation. (**A–C**) represent topographic scalp map of the EEG amplitude response for three classes of stimuli: INNO, MOD and NOX stimulation, respectively, using the average of all trials for each condition. All topographic maps were plotted for the first second post-stimulation time window (average of across each condition’s trials). The get_peak algorithm in the MNE software^22^ was used to compute and detect the amplitude of the maximum EEG response (local maxima) for the noxious stimulation as well as the location (EEG channel) and latency of the detected peak amplitude. Time courses of NOX stimulation identified and found using the get_peak algorithm were used thereafter for analysis and benchmarking with the other two conditions. Early activation of the parietal lobe was found for all conditions, followed by strong activation of the central cortex during the NOX stimulation, strong continuous activation of the parietal region during the MOD stimulus and almost no activity during the INNO stimulus. For all three conditions, no activation of the different brain regions was found when analyzing the half-second pre-stimulus phase, indicating that the subject was not anticipating the applied stimulus.

Sensory feedback of the three conditions (NOX, MOD, and INNO) tactile stimuli was delivered to the phantom hand using TENS on the transhumeral amputee’s residual limb. Three different stimulation sites were used on the residual limb, which activated the thumb/index, wrist, and pinky of the phantom hand. The NOX stimulation was reported by the subject as uncomfortable but tolerable^7^, the MOD stimulation was reported as slightly more noticeable/intense compared to the INNO stimulation. EEG was used to quantify brain activity during each stimulation and to identify the activated brain regions as well as the physiological principles underlying the perception of pain in amputees. For that, brain activity topography and time course were performed by analyzing all electrodes’ activities in the first second post-stimulus period. Our analysis shows that all stimulation conditions elicit early activation of the parietal lobe (around 54 ms) which persists over time for all types of sensation. Interestingly, the MOD stimulation elicits higher activation of the parietal lobe compared to the INNO and the NOX stimulations. In contrast to both INNO and MOD stimulations, only the NOX stimulation activates the central cortex. Based on our findings, we postulate that the NOX stimulation started in the parietal (54 ms) and centro-parietal lobe, rapidly activated the perceptual mechanism in the subject’s brain but moved thereafter towards the pre-motor and central cortex, which could, therefore, explain that the NOX stimulation activated the pain perception and reaction mechanism in the brain and the amputee had the intention to move away his residual limb during the stimulation. Results of brain activity topography for the three types of stimuli are illustrated in Fig. 2. As shown in Fig. 2, deactivations of the ipsilateral centro-parietal and the frontal lobe were observed during the NOX and the MOD stimulation, respectively, whereas a slight deactivation of the right temporal lobe was observed during the INNO stimulation. Further, 2 shows that there is no activation of the different brain regions when analyzing the pre-stimulus activation for the three different conditions, concluding that the participant was not anticipating any of the three stimuli prior to the stimulation.

### Spatio-temporal biomarkers for noxious-evoked activity

**Figure 3:**
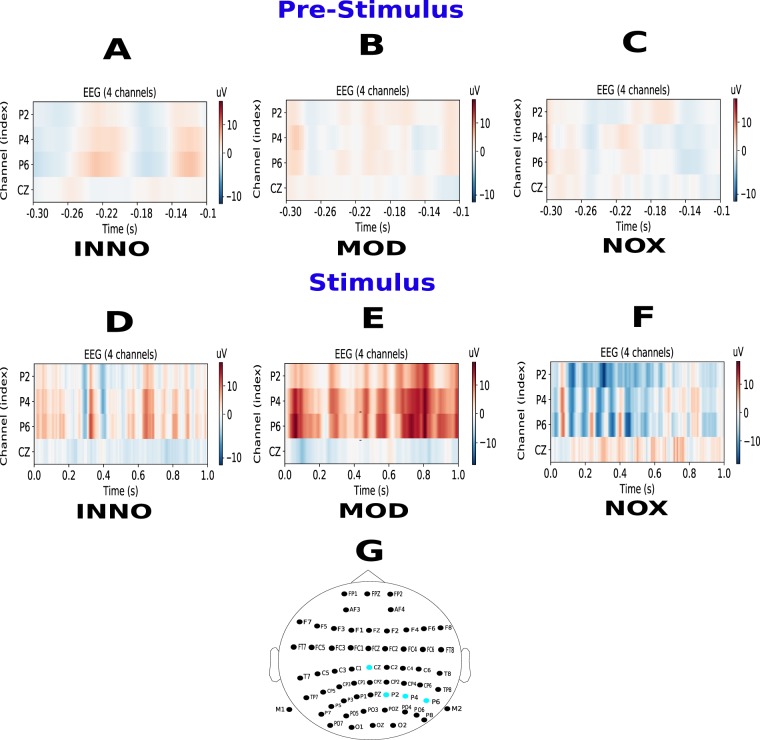
EEG activity for the parietal and central cortex electrodes. (**A–C**) represent EEG activity in Cz, P2, P4 and P6 during the pre-stimulus phase during the INNO, MOD, and NOX stimulation, respectively. (**D–F**) represent EEG activity in four different electrodes; Cz (middle cortex) and P2, P4 and P6 electrodes in the parietal lobe during the INNO, MOD, and NOX stimulation, respectively. When comparing (**D**) to (**E**), a parietal enhancement (red color) and a central depression (blue color) are observable. When comparing (**D–F**), a central enhancement is observable (red color). (**G**) 10–20 EEG recording system where Cz, P2, P4, and P6 electrode’s positions in the 10–20 system are highlighted in blue.

We then extended the aforementioned analysis by seeking to identify a spatio-temporal template to distinguish between the three conditions and find the exact brain response time and spatial location. For the NOX stimulation, the highest activation was found at the central cortex in the post-stimulation time window from 450 to 750 ms when comparing it to the INNO stimulation, and the EEG background activity [p-value < 0.0001, Kruskal-Wallis test was combined with the post-hoc (using Tukey HSD test) for multiple group comparison. Number of trials = 60]. Additionally and as shown in Fig. 3, the highest response in the amputee’s brain activity during the noxious stimulation was localized at the vertex electrode in the middle of the scalp (Cz) [p-value < 0.001, when comparing to the other two conditions. Kruskal-Wallis test was combined with the post-hoc (using Tukey HSD test) for multiple group comparison. Number of trials = 60] (relatively high activation was also detected in C4 and C6 electrodes). In contrast with the NOX stimulation which shows high activation of the central cortex, the MOD stimulation was found to be high at P2, P4, and P6 electrodes for the whole second of analysis as depicted in Fig. 3 [p-value < 0.05, Kruskal-Wallis test was combined with the post-hoc (using Tukey HSD test) for multiple group comparison. Number of trials = 60]. Although an activation of the parietal lobe was also found during the INNO stimulation, it should be noted that no special spatio-temporal biomarkers were observed. Based on our findings, we postulate that the intense stimulation was perceivable by the subject, which led to high and continuous activation of the parietal lobe in the brain^17^ but the level of discomfort was not high to activate the central and pre-motor cortex, which is responsible for motor movements. Figure 3 illustrates activation of the parietal and centro-parietal cortex for the different conditions. Similarly and as was shown in Fig. 2, no pre-stimulus activation was detected in the parietal lobe (P2, P4, and P6 electrodes) nor in the central cortex (Cz) for the three different stimulation conditions.

### Successful classification of the three different stimulation conditions

**Figure 4:**
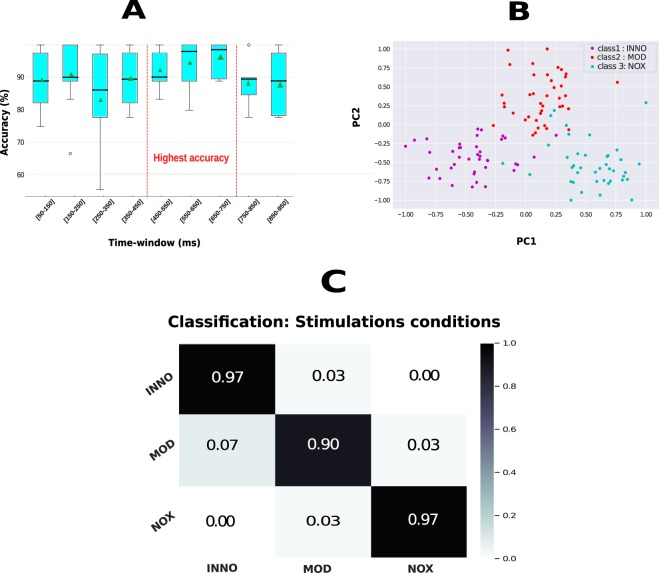
Classification results of the three stimulation conditions. (**A**) Validation accuracy in different time-windows between 50 and 1000 ms after stimulation represented in a boxplot, and showing that the highest validation accuracy was obtained in the time-window 650–750 ms. The green triangle represents the mean accuracy value for each time window whereas the black line represents the median value for the same time window. (**B**) 2D feature space after performing PCA, highlighting a clear separation between the three conditions. PC1 and PC2 represent the first two components after performing PCA. (**C**) As the highest accuracy was obtained between 650–750 ms (shown in **A**), the confusion matrix was computed in that time-window when classifying the three stimulation conditions in the test phase.

**Figure 5:**
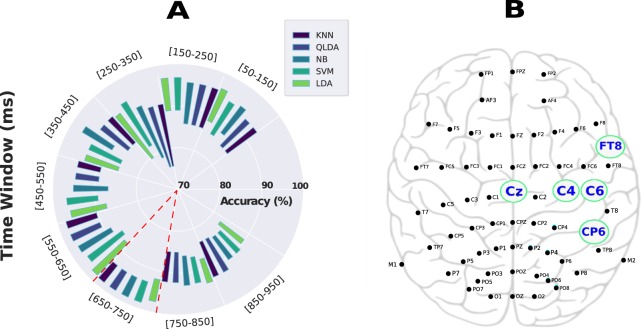
Validation results of the classification of the three stimulation conditions in each time-window using five different classifiers (KNN:K-Nearest Neighbors, QLDA:Quadratic Discriminant Analysis, NB:Naïve Bayes, SVM:Support Vector Machine, LDA:Linear Discriminant Analysis) as well as the selected electrodes using the feature sequential algorithm. (**A**) Validation accuracy in different time-windows between 50 and 1000 ms after stimulation using five different classifiers and represented with a polar bar plot. The polar bar plot shows the accuracy range (mean standard deviation) achieved by the five models for each time-window. The highest accuracy was obtained in the time-window [650–750] and is highlighted in the figure in red. (**B**) The 10/20 system used for EEG data recording. A feature selection algorithm selected five different features (Cz, C4, C6, CP6, and FT8 electrodes) when classifying the three different conditions. Their positions in the brain, according to the 10/20 system, are highlighted in (**B**).

We further investigated EEG based spatio-temporal biomarkers for the classification of the three different stimulation conditions. First, we studied how the classification accuracy changes in each 100 ms post-stimulation time. As shown in Fig. 4(A), our findings are in accordance with our aforementioned analysis results, and higher mean balanced accuracy (bACC) was found in the time window between 450 and 750 ms, with the highest bACC found between 650 and 750 ms post-stimulation [p-value < 0.005, Kruskal-Wallis test was combined with the post-hoc (using Tukey HSD test) for multiple group comparison. Number of trials = 60]. We wish to emphasize that a mean accuracy of more than 90% was also observed in the time-window between 150–250 ms, which could be also further investigated and used as a time marker to design a nociceptive pain reaction system, where a minimum delay is usually required^23^. As the highest validation accuracy (validation bACC) was found in the time-window 650–750 ms, we used that time window during the test phase to classify the three different conditions and for further analysis. A mean bACC of 95% was obtained in the validation phase and more than 94% was achieved in the test phase, whereas the chance level for this task is 33.33%. For that, data were split into 80% and 20% where stratified 10-fold cross-validation was performed on 80% of the data (validation phase) and the saved model was used to predict correct labels on the remaining 20% of the trials in the test phase. A principal component analysis (PCA) was performed on the recorded data, which resulted in a clear separation between the three classes as shown in Fig. 4(B). Figure 4(C) presents the obtained results in the test phase. The confusion matrix shows that both INNO and NOX stimulation were almost correctly classified, whereas the MOD stimulation was sometimes either confused with the INNO or the NOX stimulation. Last, it is important to highlight that when we kept all the features (without performing dimensionality reduction using PCA), we observed that our feature selection algorithm selected Cz, C4, C6, FT8 and CP6 as the best electrodes/features when distinguishing between the three classes. Selected electrodes are shown in Fig. 5 and are well aligned with our aforementioned analysis, where we showed that central and the centro-parietal cortex electrodes exhibited different activities depending on the stimulation condition, and hence of the utmost importance when differentiating between the different types of stimulation. It is worth noting that the computed features are simply the maximum amplitude value of each electrode, which led to a feature matrix of 64 dimensions.

### Noxious-related activation within the medial wall of the cerebral cortex

**Figure 6:**
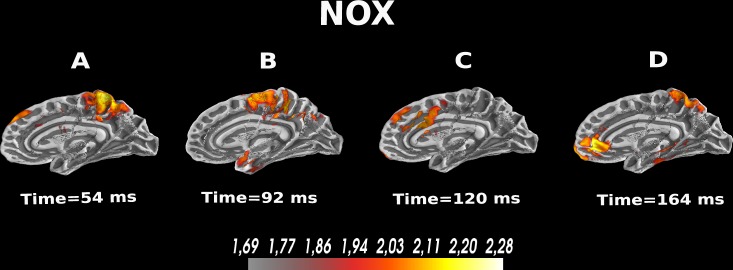
EEG analysis at the source level for the noxious evoked activity in the first 200 ms. The dynamic statistical parametric maps (dSPM)^30^ was used to compute the reconstructed sources. The used scale represents the EEG amplitude activity in uV. (**A**) High EEG activity in the centro-parietal lobe after 54 ms of stimulation. (**B**) High EEG activity in the central cortex after 92 ms. (**C**) Activation of the PCC after 120 ms. (**D**) Activation of the ACC and the parietal lobe after 164 ms.

To extend our findings, we sought to study brain activity at the source level in response to NOX, MOD, and INNO stimulations, and gain more insights into how the pain reaction mechanism is achieved in the brain and what are the different brain regions involved in this pain reaction circuitry. We identify activated brain sources, en route to developing a complete real-time pain sensation detection system. We focus our analysis mainly on the NOX stimulation, which triggers the reaction mechanism in the brain. Here, we investigate if scalp EEG can detect and localize signals originating from different brain areas during the three different conditions. Overall, our analysis confirms the evidence that scalp EEG could be used to detect and correctly localize the source of the recorded signals^24^. For that, we reconstructed EEG source dynamics using distributed source modeling^25,26^ based on realistic head models^27^ derived from individual MRI scans^24^. As illustrated in Fig. 6, we found using the reconstructed EEG sources that NOX sensation elicits activation of the centro-parietal lobe, activation of the anterior cingulate cortex (ACC), the somatosensory motor cortex, and the posterior cingulate cortex (PCC). Overall and as shown in Fig. 6, the activation of the ACC presents direct evidence that it plays a role in activating the attention circuitry in the brain as well as an important role in external sensory stimuli perception^28,29^. Moreover, our study reveals that the NOX stimulation activates the PCC, which seems to be in accordance with a similar study using another sensory feedback modality^11^. As shown in Figure 1 in the Supplementary Materials, the source localisation results for the MOD and INNO conditions show also activation of the parietal lobe at around 54 ms, which is aligned with topographic maps illustrated in Fig. 2.

## Discussion

Here, a spatio-temporal template of brain activity during three different phantom limb stimulation conditions (INNO, MOD and NOX) has been identified and characterized in an amputee based on EEG signals. Unlike fMRI or intracranial signals, EEG allows continuous and real-time recording with a good compromise between level of invasiveness and temporal resolution^31^, which could be therefore used when developing real-time systems based on neural response to sensory stimulation. The findings in this study confirm and extend previous findings in non-amputee related studies on the high activation of the somatosensory and motor cortex during the NOX stimulation. Specifically, the highest activation was detected in Cz electrode site and its neighboring electrodes C4, C6 and CP6. Additionally, we also show early activation of the centro-parietal lobe for all the three conditions with the highest activity observed when perceiving and processing the MOD stimulation. Further, we identified a spatial template of noxious-evoked activity and we investigated different activated regions at the source level using EEG signals. This has overall confirmed the important role of the ACC to trigger the attention mechanism in the brain^11,32^, as well as the role of PCC for noxious stimulation processing^11^. Our study is extending upon Osborn et al. study^7^, where a pain reflex mechanism was implemented at the level of the prosthesis. Our study suggests that such a withdrawal/pain reaction system could be implemented by harnessing the neural response to painful stimuli. The withdrawal reaction could be activated when the prosthesis touches sharp objects, and hence protect it from being damaged. Along the same lines, this can help better monitor the user’s perception of what is happening to create more natural prostheses and provide the amputee more intuitive control over his prosthesis as well as increase the sense of embodiment and ownership.

### Noxious-evoked activity spatio-temporal template interpretation

Noxious-evoked activation generated high activity around Cz electrode between 450 ms–750 ms with the highest activity detected at Cz. We also investigate the relation between brain responses and three types of sensations. Based on that, we could distinguish between the three different conditions with more than 94% accuracy using an identified spatio-temporal template based on a subset of electrodes and the temporal markers. Similarly, we studied the temporal latency for each condition as well as the spatial distribution of the activity across other electrode sites. Even though exact mechanisms responsible for the delay remain to be investigated, we postulate that the early activation of the parietal lobe during all conditions makes strong evidence on its role in early processing of relevant somatosensory stimuli and its heavy connection with the ACC in painful and intense stimuli processing forming a complete circuitry for somatosensory stimuli detection and perception. We also hypothesize that only the NOX stimulation triggers the pain reaction mechanism in the brain, reflecting one’s intention to escape the potential source of pain, which relies on the motor cortex. However, the processing of the other two conditions remain at the parietal lobe level and did not activate any motor reaction. The important role of the ACC, PCC, and the pre-motor cortex in noxious sensation perception in amputees was also investigated during the noxious sensation. Thereafter, we observe activation of the motor cortex, the ACC, and the PCC, which is thought to be along the descending pathway to the spinal cord. Overall, our source localization results present new evidence on the similarity of the reflex system in amputees and healthy subjects. Further, our study reveals a high correlation between the stimulation condition and the activation magnitude of the ACC, which is aligned with previous studies for other purposes. Interestingly, all conditions activated the anterior portion of the ACC, which is known to be engaged in different attention networks in the brain as well as sensory stimuli processing and were reported in different clinical pain neuroimaging studies. Further, high activation of the PCC was observed during the NOX stimulation. Overall, it has previously been shown that the PCC is part of motor responses with a wide interaction with the parietal lobe. In fact, PCC is adjacent to the supplementary motor cortex (SMA), which could explain why SMA activation was only shown during the noxious stimulation.

### Towards a real-time reflex system in prostheses

Beyond the scope of this work, the ultimate goal of this research is to design and develop a system that can detect in real-time pain reaction in the amputee’s brain and use that detection to trigger a withdraw reaction in the prosthesis, not only to protect it from being damaged but also to increase the amputee’s sense of ownership and mimic the natural closed-loop control of upper-limb in healthy subjects. The lack of such a closed-loop control system is so far one of the main causes of prosthesis rejection and abandonment^33^. Aside from analyzing and understanding the underlying mechanisms of different stimuli processing in the brain during sensory feedback in prostheses, we successfully classified the three different conditions with more than 94% accuracy based on the aforementioned findings. Despite the good obtained accuracy, one of the limitations of this study is that all analyses were obtained from one amputee. We overcome this limitation by showing that most of these findings have been validated before in other studies with different purposes and goals. Additionally, investigating brain reaction to external sensory stimuli and presenting a spatio-temporal brain template to study NOX, MOD and INNO stimulation effects using EEG may slightly differ depending on the circumstances and parameters, such as genetic and environmental differences, age and gender, as well as time since amputation, which present evidences that can be only generalized to some extent across multiple amputees.

## Methods

### Patient recruitment and sensory stimulation

An amputee participant (29 years old) with a bilateral amputation for more than five-year prior to the current experiments, due to tissue necrosis from septicemia, was recruited at Johns Hopkins University in Baltimore to perform a series of an embodied prosthesis control as well as sensory feedback experiments. The participant has a transhumeral amputation of the left arm and a transradial amputation of the right arm. All sensory feedback and prosthesis experiments were performed on the participant’s left arm. EEG data were collected in one session over a period of two hours. For the sensory stimulation, we performed sensory mapping of the amputee’s phantom hand through transcutaneous electrical nerve stimulation (TENS) using a 1-mm beryllium copper (BeCu) probe connected to an isolated current stimulator (DS3, Digitimer Ltd., Hertfordshire, UK). An amplitude of 0.8 mA and frequency of 2 to 4Hz were used while mapping the phantom hand. The amputee identified areas of phantom activation during sensory mapping and the stimulation sites were noted using anatomical and ink markers. For the stimulation experiment, we used 5-mm disposable Ag-Ag/Cl electrodes on the residual limb sites that mapped to the thumb/pointer, pinky/ulnar, and wrist of the phantom hand^34^. The stimulation sites were the same as those used in^7^. It should be noted that sensory mapping was only performed on the left (transhumeral) residual limb because the amputee participant only wears a prosthesis on his left (transhumeral) side and not his right (transradial) side.

### Research governance

This study was carried out in accordance with the Declaration of Helsinki. All experiments were approved by the Johns Hopkins Medicine Institutional Review Boards. The participant was asked to sign a written informed consent and he agreed to take part in all our experiments. Additionally, the participant consented, by signing a written informed document, to have images and recordings taken during the experiments used for online open-access publication and presentations.

### EEG data recording and experiment

Brain activity correlates of transcutaneous electrical nerve stimulations were investigated by recording 64-channel EEG data from the amputee participant. Different locations on the participant’s left residual limb were identified so that, when stimulated, they activate different regions of the participant’s phantom hand. In this EEG experiment, the subject was seated comfortably and was looking at a black cross on a white wall. EEG recordings during various stimulations of the subject’s peripheral nerve sites corresponding to the thumb/pointer finger, pinky/ulnar side of the hand, and the wrist of his phantom hand. We stimulated the subject’s residual limb in regions that activated his phantom hand using transcutaneous electrical nerve stimulation (TENS). The stimulation included three different conditions for the thumb/pointer and two conditions for the other sites. All values of stimulation were based on previous mapping and psychophysics with this subject^7^. Condition 1 (INNO stimulation): Pulse width (PW) = 1 ms, freq = 45 Hz - perceived as a light, almost pleasant touch sensation. Condition 2 (MOD stimulation): PW = 5 ms, freq = 4 Hz - perceived as a slightly more noticeable/intense touch but not uncomfortable. Condition 3 (NOX stimulation): PW = 20 ms, freq = 20 Hz - perceived as a slightly painful and uncomfortable touch sensation. All values of stimulation were based on previous mapping and psychophysics with this subject^7^. All three conditions (INNO, MOD, NOX) were applied to the thumb/pointer stimulation site and the INNO and MOD conditions were applied to the pinky/ulnar and wrist stimulation sites. Blocks of each stimulation condition were randomly presented as a five consecutive stimulation pulse trains lasting for 2 s with a delay of 4 s $\pm$ 25% jitter between each stimulation pulse train. Stimulation condition blocks were presented 4 times, yielding a total of 60 trials for the three conditions. A break of 2 min was given between stimulation blocks, and a break of 10 min was given between the different stimulation sites. Condition 3 (NOX) was only presented to the thumb/pointer stimulation site, whereas Conditions 1 and 2 (INNO and MOD) were presented to all stimulation locations (thumb/pointer, pinky/ulnar, and wrist). Condition 3 was only presented to the thumb/pointer location to reduce the total time the subject experience noxious sensations. EEG data were collected using a 64 channel EEG device (Neuroscan system) with a 500 Hz sampling rate. The montage was in accordance with the 5% 10/20 system. Electrode impedance was kept below 10 kOhm in at least 95% of derivations throughout the experiment. The amplitude of the transcutaneous electrical nerve stimulation was 1.6 mA for all sites of stimulation and the subject rated each condition’s discomfort level using a comfort scale. To ensure that the subject is not substituting/anticipating the stimuli by sight, the EEG data was recorded without the subject wearing the prosthesis. The pulse width and the frequency of the TENS stimulation were selected based on extensive psychological experiments to quantify the perception of TENS as was reported in^7,34^.

### EEG signal processing and classification

EEG data were recorded at 500 Hz. The reference electrode was chosen on the vertex and the ground electrode was located on the forehead. Data were processed with special designed Jupyter notebooks in Python using both gumpy^35^ and MNE^22,36^ toolboxes. For data analysis, 60 trials in total for the three stimulation conditions were used. EEG signals were band-pass-filtered between 0.5 and 70 Hz using a fourth-order Butterworth filter and notch filtered thereafter at 60 Hz^12,37^. All signals were extracted from the recordings in 1000 ms epochs, and to 100 ms time-window for further analysis. Epochs were baseline-corrected to the pre-stimulus mean^38^. Muscle artifacts were rejected by the Automatic Artifact Rejection (AAR)^39^ as well as independent component analysis (ICA) were used to remove eye movement artifacts^40^. Additionally, epochs containing high-amplitude artifacts or high-frequency muscle noise (visually inspected) were rejected from the analysis using a threshold-based method^41^. EEG data collected over several trials of the same experiment were averaged together. All EEG scalp topographies were plotted using the MNE toolbox, by matching channel location with its value given the defined latency. Topographies are color encoded, where the green or yellow present null values, blue color presents negative values, and red encodes positive values; with the color intensity correlates with the channel value. Chosen time latencies in the topographic maps were chosen based on an algorithm^22^ that computes and finds the highest peaks at each time point from all electrodes. For feature extraction and classifying the three conditions from EEG, we implemented and tested a wide range of classical machine learning approaches which are based on hand-crafted features. Five different classifiers from the gumpy.classification module^35^ have been used and evaluated: K-Nearest Neighbor (KNN), Support vector machine (SVM), Naive Bayes (NB), Linear Discrimination Analysis (LDA) and Quadratic Linear Discrimination Analysis (QLDA). Two different feature extraction methods were used, namely the maximum amplitude value computed from each channel for a fixed 100 ms time-window, yielding a total number of 64 features (number of electrodes) as well as common spatial patterns (CSP)^42^. The CSP method yielded to slightly lower results (a mean accuracy of 85%) than the maximum amplitude value and was therefore discarded in our further analysis. Two different post processing methods were investigated and tested. First, a principal component analysis (PCA) with only two components was used for dimensionality reduction and the two components were fed thereafter to the different classifiers resulting in the presented 2D feature space (Fig. 4). Second, we further investigated keeping all the extracted 64 features and we used a feature selection algorithm^43^ to select the most discriminating subset of features (channels) as was depicted in Fig. 5. Data were divided into 80% for training and 20% for testing. As the total number of 60 trials was small when splitting the data into validation and test sets (solely four trials remained for each condition during the test phase), a data augmentation was performed^37^. This yielded two separate trials from each one of the initial 60 trials (by splitting a 2-sec trial into 2 segments of 1-sec length each), thus forming a total number of 120 trials. It should be mentioned that the 120 trials (after data augmentation) were only used when performing the PCA, splitting the data, and computing the test accuracy. Overall, a 10-fold cross validation was performed on training data to validate the model (validation accuracy) and the remaining 20% were using for the test phase. It should be noted for all analyses, balanced accuracy (bACC) was chosen as an evaluation metric for the trained models. bACC is calculated as the average of the proportion corrects of each class individually, where the same number of examples in each class was used. Overall, we wish to mention that the first feature extraction method (max amplitude value) combined with PCA using SVM clearly outperformed the other investigated methods, yielding a validation accuracy of more than 95% and a test accuracy of more than 94%. It is worth noting that a grid search was performed to select the best hyper parameters of the SVM classifier for a given 10-fold cross validation.

### GFP computation

**Figure 7:**
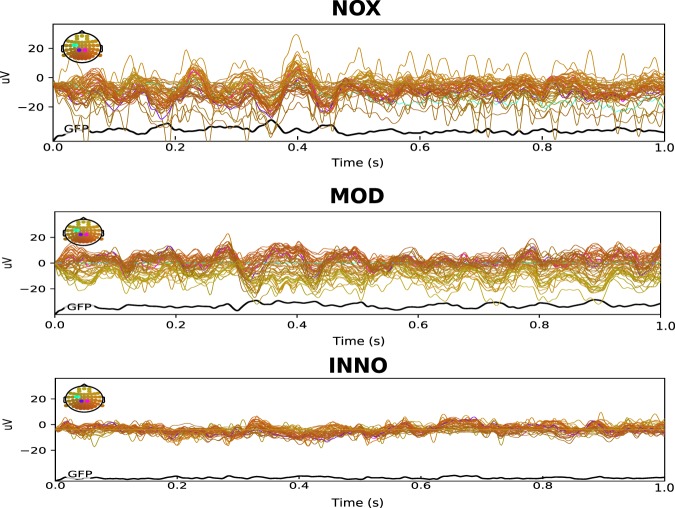
EEG activity for all 64 electrodes combined with the global field power (GPF) shown for the three different stimulation conditions. NOX stimulation shows higher GFP than the other two conditions and high activity in the somatosensory electrodes (CZ, C4, and C6). MOD shows higher GFP than the INNO and higher activity in the parietal lobe electrodes (P2, P4, and P6). Low GFP is shown during the INNO stimulation. The electrodes are color-coded as shown in the 10/20 system in the figure (up-left).

The Global Field Power (GFP) is the standard deviation of the potentials at all EEG channels of an average given reference map^44^. The GFP formula is shown below in equation (1):

1 $$GFP=\frac{\sqrt{{\sum }_{i=0}^{{\rm{N}}}({{\rm{\mu }}}_{i}-{\bar{\mu }}^{2})}}{N}$$

where ${\mu }_{i}$ is the voltage of the map $\mu$ for a given electrode i, $\bar{{\rm{\mu }}}$ is the mean voltage of all EEG electrodes of the map u and N is the number of electrodes of the map $\mu$. High GFP wis explained by peak EEG activities as well as steep gradients. Overall, as illustrated in Fig. 7, NOX stimulation shows higher global field power (GFP) compared to the MOD and the INNO stimulation. The GFP shows here the amount of activity at each time point in the field considering the data from all the 64 recording electrodes simultaneously^45^.

### Source localization

MNE toolbox^22^ combined with gumpy^35^ Python toolbox were used for EEG processing and for source localization. First, cortical surface reconstruction using FreeSurfer^46^. Second, the forward solution and the forward model were computed using the boundary-element model (BEM)^47^. Thereafter, the regularized noise-covariance matrix, which gives information about potential patterns describing uninteresting noise source, was computed and estimated. Afterward, we computed the singular value decomposition (SVD) of the matrix composed of both estimated noise-covariance and the source covariance matrix. Finally, dynamic statistical parametric maps (dSPM)^30^ was computed and used for source localization and reconstruction. For dSPM, an anatomical linear estimation approach is applied. This assumes the sources are distributed in the cerebral cortex^48^. A linear collocation single-layer boundary-element method (BEM)^49^ is used to compute the forward solution which models the generated signal pattern at each location of the cortical surface. A noise-normalized minimum norm estimate is estimated at each cortical location resulting in an F-distributed estimation of the cortical current. Overall, dSPM identifies the locations of statistically increased current-dipolar strength relative to the noise level. The sLORETA method for source localization was also implemented for benchmarking purposes and is available in the Supplementary Materials.

### Data analysis and statistics

All relevant information about the obtained results is presented alongside their corresponding figures. For all statistical analyses, we first checked the normality as well as the independence of the data before applying the adequate statistical test. The normality of the data (the three conditions with the background activity) was checked using one-sample Kolmogorov-Smirnov test, which is a strict normality test, and as was suggested in the study by Strauss *et al*.^50^. Thereafter, the independence was checked using the Mann-Whitney U Test was used (used when data is not normally distributed) and we found that the conditions are statistically independent (p-value < 0.0001). As the data was not normally distributed, we applied a Kruskal-Wallis test instead of ANOVA^50^. The Kruskal-Wallis test was combined with the post-hoc test for multiple group comparison. For all the obtained results, we considered p-value < 0.05 statistically significant to reject the null hypothesis. The software for the statistical analysis was implemented in python using the scipy and stats libraries.

### Accession codes

All software codes will be made publicly available at https://github.com/gumpy-bci/gumpy.

## Supplementary information

Supplementary Information 1.

Supplementary Information 2.
