# Supplementary material for: Decoding of Pain Perception using EEG Signals for a Real-Time Reflex System in Prostheses: A Case Study
Source: Sci Rep. 2020 Mar 27;10:5606. doi: 10.1038/s41598-020-62525-7 (PMC7101312; doi:10.1038/s41598-020-62525-7)
Supplement: Supplementary file 2 — Supplementary Information 2. [file 41598_2020_62525_MOESM2_ESM.pdf]

Supplementary materials for: Decoding of Pain Perception using EEG Signals for a Real-Time Reflex System in Prostheses: A Case Study

Zied Tayeb[1,*], Rohit Bose[2, 3], Andrei Dragomir[2, 4], Luke E. Osborn[5, 6], Nitish V. Thakor[5, 7], and Gordon Cheng[1]

[1]Institute for Cognitive Systems, Technical University of Munich, Arcisstraße 21, 80333 München, Germany
[2]N.1 Institute for Health, National University of Singapore, 28 Medical Dr. 05-COR, Singapore 117456, Singapore
[3]Department of Bioengineering, University of Pittsburgh, 3700 O'Hara Street, Pittsburgh, PA 15261, USA
[4]Department of Biomedical Engineering, University of Houston, 3517 Cullen Blvd, Houston, TX 77204, USA
[5]Department of Biomedical Engineering, Johns Hopkins School of Medicine, 720 Rutland Ave, Baltimore, MD 21205, USA
[6]Research Exploratory Development, Johns Hopkins University Applied Physics Laboratory, 11100 Johns Hopkins Rd, Laurel, MD 20723, USA
[7]Department of Biomedical Engineering, National University of Singapore, Engineering Drive 3, 04-08, Singapore 117583
[*]zied.tayeb@tum.de

Standardized LORETA (sLORETA) method

The Standardized low-resolution brain electromagnetic tomography (sLORETA)[1] computes the EEG distribution across the full brain volume. The sLORETA method provides a smooth and good deep sources localization. sLORETA provides a unique solution to the inverse problem using a defined cost function[2] F as follows:

$$F = \|\phi - KJ - c1\|^2 + \alpha \|J\| \tag{1}$$

where ϕ defines the electrical potential recorded from the 64-electrode scalp EEG signal, K defines the lead field matrix, J represents the measured current density, α defines the positive regularization parameter, $c1$ is constant, and $\|.\|$ is the Euclidean norm. For benchmarking purposes, the sLORETA was also implemented and used for EEG source localisation. As illustrated in Figure 1 and was previously reported in[3], results obtained by the sLORETA method are similar and match the obtained results by the dSPM method with a slightly lower values when using the sLORETA technique[3].

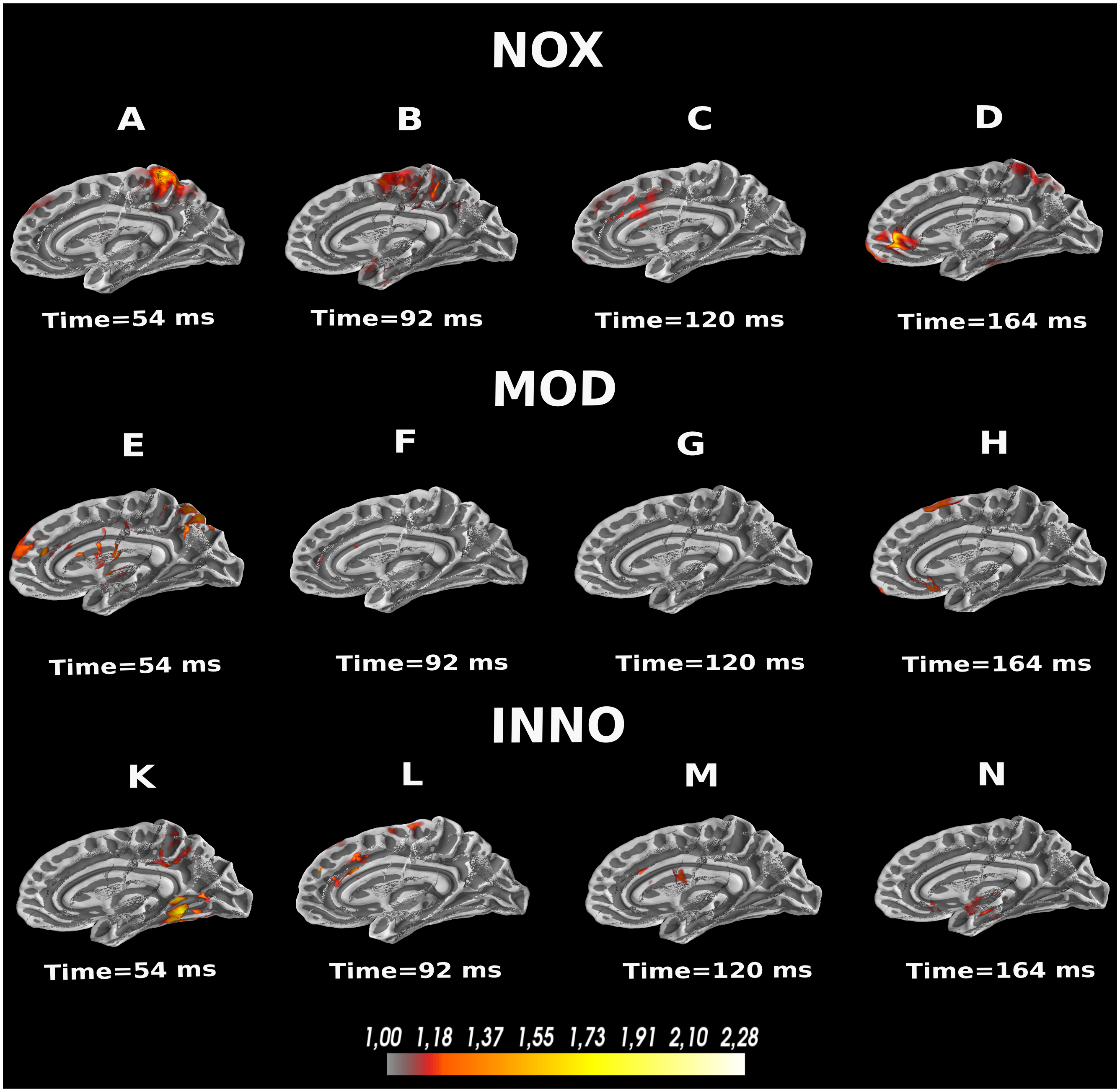

Figure 1. EEG analysis at the source level for the different conditions in the first 200 ms using sLORETA. The sLORETA method was computed and used for benchmarking with the dSPM method. Results from both methods are similar with lower values were found for sLORETA. The used scale represents the EEG amplitude activity in uV. **A**. High EEG activity in the centro-parietal lobe after 54 ms of stimulation. **B**. High EEG activity in the central cortex after 92 ms. **C**. Activation of the PCC after 120 ms. **D**. Activation of the ACC and the parietal lobe after 164 ms. **E**. EEG activity in the centro-parietal lobe after 54 ms of stimulation. **F**. No high EEG activity was detected after 92 ms of stimulation. **G**. No high EEG activity was detected after 120 ms of stimulation. **H**. No high EEG activity was detected after 164 ms of stimulation. **K**. EEG activity in the centro-parietal lobe after 54 ms of stimulation. **L**. No significant EEG activity was detected after 92 ms of stimulation. **M**. No significant EEG activity was detected after 120 ms of stimulation. **N**. No significant EEG activity was detected after 164 ms of stimulation.

References

1. Pascual-Marqui, R. Standardized low resolution brain electromagnetic tomography (SLORETA): Technical details. *Methods findings experimental clinical pharmacology* **24 Suppl D**, 5–12 (2002).

2. Jatoi, M., Kamel, N., Malik, A. & Faye, I. EEG based brain source localization comparison of sLORETA and eLORETA. *Australas. Phys. Eng. Sci. Medicine* **37** (2014).

3. Hauk, O., Wakeman, D. & Henson, R. Comparison of noise-normalized minimum norm estimates for MEG analysis using multiple resolution metrics. *NeuroImage* **54**, 1966–74 (2010).
